# Diagnoses and Treatment Recommendations—Interrater Reliability of Uroflowmetry in People with Multiple Sclerosis

**DOI:** 10.3390/biomedicines12071598

**Published:** 2024-07-18

**Authors:** Anke K. Jaekel, Julia Rieger, Anna-Lena Butscher, Sandra Möhr, Oliver Schindler, Fabian Queissert, Aybike Hofmann, Paul Schmidt, Ruth Kirschner-Hermanns, Stephanie C. Knüpfer

**Affiliations:** 1 Clinic for Urology, University Hospital Bonn, 53127 Bonn, Germanyrkirschnerhermanns@googlemail.com (R.K.-H.); stephanie.knuepfer@ukbonn.de (S.C.K.); 2 Department of Neuro-Urology, Johanniter Rehabilitation Centre Godeshoehe, 53177 Bonn, Germany; 3 Clinic for Neurorehabilitation and Paraplegiology, REHAB Basel, 4055 Basel, Switzerland; s.moehr@rehab.ch; 4 Clinic for Urology, University Hospital Ulm, 89070 Ulm, Germany; 5 Clinic for Urology, University Hospital Münster, 48149 Münster, Germany; 6 Clinic St. Hedwig, Department of Paediatric Urology, University Medical Center Regensburg, 93053 Regensburg, Germany; 7 Statistical Consulting for Science and Research, Berlin Statistical Consulting for Science and Research, 13086 Berlin, Germany

**Keywords:** multiple sclerosis, bladder disorder, neurogenic, bladder

## Abstract

Background: Uroflowmetry (UF) is an established procedure in urology and is recommended before further investigations of neurogenic lower urinary tract dysfunction (NLUTD). Some authors even consider using UF instead of urodynamics (UD). Studies on the interrater reliability of UF regarding treatment recommendations are rare, and there are no relevant data on people with multiple sclerosis (PwMS). The aim of this study was to investigate the interrater reliability (IRR) of UF concerning diagnosis and therapy in PwMS prospectively. Methods: UF of 92 PwMS were assessed by 4 raters. The diagnostic criteria were normal findings (NFs), detrusor overactivity (DO), detrusor underactivity (DU), detrusor–sphincter dyssynergia (DSD) and bladder outlet obstruction (BOO). The possible treatment criteria were as follows: no treatment (NO), catheter placement (CAT), alpha-blockers, detrusor-attenuating medication, botulinum toxin (BTX), neuromodulation (NM), and physiotherapy/biofeedback (P/BF). IRR was assessed by kappa (κ). Results: κ of diagnoses were NFs = 0.22; DO = 0.17; DU = 0.07; DSD = 0.14; and BOO = 0.18. For therapies, the highest κ was BTX = 0.71, NO = 0.38 and CAT = 0.44. Conclusions: There is a high influence of the individual rater. UD should be subject to the same analysis and a comparison should be made between UD and UF. This may have implications for the value of UF in the neuro-urological management of PwMS, although at present UD remains the gold standard for the diagnostics of NLUTD in PwMS.

## 1. Introduction

Uroflowmetry (UF) has been a well-established diagnostic procedure in urology for many years [1]. It is used to diagnose functional disorders of the lower urinary tract in children and adults [2,3]. In this non-invasive urodynamic procedure, a urinary flow rate per time is determined using various technical principles and a flow curve is recorded while voiding urine [4]. Finally, the post-void residual (PVR) is measured by sonography of the bladder [5]. Conclusions can be drawn about the existing type of anatomical or functional disorders of the lower urinary tract from the various forms of UF curves, the micturition volume, the strength of the urine flow, and PVR [4]. The advantages of UF are its simplicity, non-invasiveness, and short duration [1].

The procedure is indicated for both neurogenic and non-neurogenic functional disorders of the lower urinary tract. It is recommended in various urological guidelines [2,3,5]. For neurogenic lower urinary tract dysfunction (NLUTD) in people with multiple sclerosis (PwMS), so far there are no explicit guideline recommendations for UF. Nevertheless, individual authors recommend uroflowmetry, even as a substitute for urodynamics [6], which is the gold standard in the diagnostics of NLUTD [7]. Others only recommend urodynamics for PwMS in the case of treatment failure or prior to surgical treatment [8]. These recommendations are based on the fact that urodynamics (UD), associated with high effort and invasiveness, have ultimately no impact on the treatment outcome. Therefore, neuro-urological expert committees discuss whether UF can play a role in reducing the need for invasive UD [9].

Recommendations for the standardised performance and reporting of uroflowmetry were published by the International Continence Society in 2002 [4]. Due to the involvement of the autonomic nervous system in micturition, there are many factors influencing uroflowmetry: the environment, the patient’s compliance and current situation, the micturition position, and the voiding volume [10,11]. Clear recommendations on how to perform UF were defined: a minimum bladder-filling volume should be maintained, the person should void in their usual posture, and information on the subjective representativeness of the examination should be obtained [4,12].

Another significant factor influencing UF is the rater-related individual interpretation of the examination. The rater develops a treatment proposal from his/her subjective perspective by the inclusion of clinical information, bladder diaries (BDs), and questionnaires [13].

The extent to which a measurement procedure is dependent on the influence of the examiner can be measured using reliability [14]. Interrater reliability (IRR) measures the agreement of the assessment between different raters. There are a variety of studies on IRR of UF in children and adults with different study designs [15,16,17]. So far, there has been no study on the IRR of UF in PwMS that relates not only to the uroflow curve itself, but also to the underlying diagnosis and treatment recommendation.

To assess the long-term significance of UF in comparison to UD regarding NLUTD in PwMS, our intention was to assess the IRR of UF regarding suspected diagnosis and treatment recommendations. We also analysed the IRR of the suspected diagnosis from medical history and BDs.

## 2. Patients and Methods

This study included all PwMS who had been presented to the neuro-urology department of an inpatient neurological rehabilitation centre for further diagnosis of NLUTD between 2017 and 2022 and who met the following requirements: written informed consent for prospective evaluation of their data, detailed urological history, a completed BD with documented drinking and micturition volumes in ml over 2 days, and uroflowmetry that was performed according to the standards of the International Continence Society. Ninety-two PwMS met these criteria. Inclusion in this study was independent of the clinical course or severity of the disease. Exclusion criteria were invalid uroflowmetry with a micturition volume of <150 mL, a BD that could not be analysed due to invalid or illegible documentation, and the absence of an informed consent form.

All UF examinations were conducted by a highly experienced neuro-urological team consisting of 2 physicians and 3 nurses. The UF was indicated after the patient had undergone an initial consultation with the physician. The examination was scheduled on a different date than the initial consultation. The UF examination procedure was explained in detail in advance. All of the participants were scheduled for UF in the same standardised time slot with a filled bladder. The examination was conducted, according to the preference of the PwMS, in a sitting or standing position in a closed quiet toilet suitable for the disabled. The measurement was performed using an MMS Nexam Pro Urodynamic System (Laborie/Medical Measurement Systems B.V., Enschede, The Netherlands) after the participants had expressed a strong desire to void urine. The measurement of PVR was performed by transabdominal sonography with a GE LOGIQ S7 Pro (GE Ultrasound Korea Inc. Seongnam-si, Korea).

**Figure 1:**
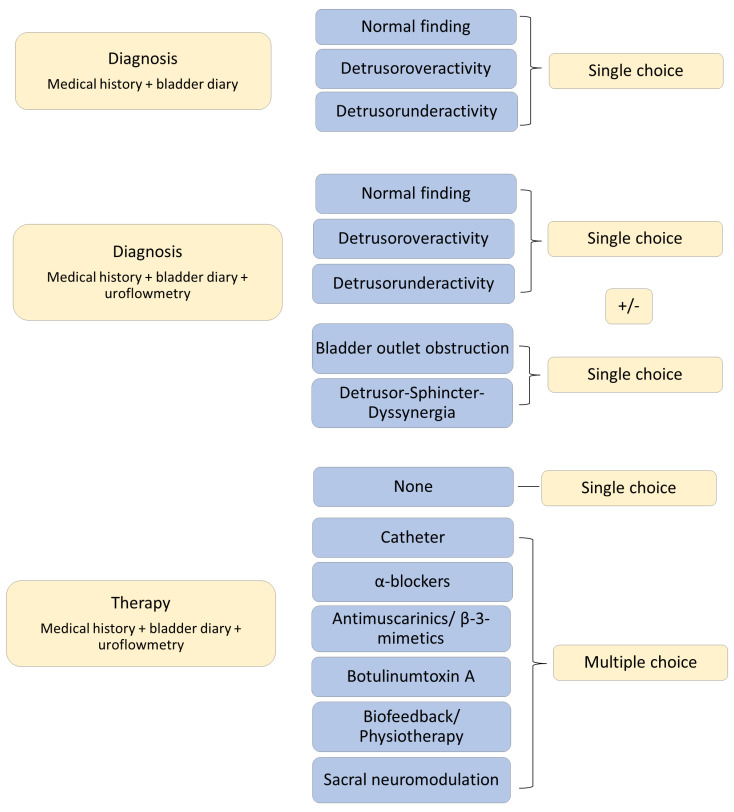
Visualisation of the potential answers on the evaluation form. “+/−“—a combination of both single choices is optional, but not mandatory.

Four experienced neuro-urologists were recruited as raters. They received the following data: diagnoses of the participants, a summarised medical history, information on gender, summarised information from BDs, graphics of the UF curves with information on voided volume in ml, PVR in ml, and maximum urinary flow in ml/s. The raters were asked to assign the individual uroflowmetry findings to the most appropriate diagnostic category, considering the clinical information and the BDs, and to select a corresponding treatment recommendation. A standardised evaluation form was used for this purpose. The answering options listed in the form are shown in Figure 1. All raters were blinded to the original clinical findings.

The primary endpoint was the IRR regarding the following items:

- (A) Suspected diagnosis from medical history and BDs;
- (B) Suspected diagnosis from medical history, BDs, and UF;
- (C) Therapy suggestion from medical history, BDs, and UF.

For all primary endpoints, the agreement between 2 raters was determined using Cohen’s kappa (κ_C_) and the agreement between all raters was assessed using Fleiss’ kappa (κ_F_). For the primary endpoints B and C, the answers could be obtained using multiple-choice assessment (see Figure 1). The possibility of multiple-choice assessment resulted in the following analyses:

**Analysis** **A:** *IRR for the suspected diagnoses from the medical history and BDs;*

**Analysis** **B-1:** *IRR for each single suspected diagnosis from medical history, BDs, and UF;*

**Analysis** **B-2:** *IRR for the combined suspected diagnoses from medical history, BDs, and UF;*

**Analysis** **C-1:** *IRR for each single therapy suggestion from medical history, BDs, and UF;*

**Analysis** **C-2:** *IRR for the combined therapy suggestions from medical history, BDs, and UF.*

On a scale of 0.0–1.0, a kappa statistic of 0–0.2 indicates slight agreement, 0.21–0.4 indicates fair agreement, 0.41–0.6 indicates moderate agreement, 0.61–0.8 indicates substantial agreement, and 0.81–1.0 indicates nearly perfect agreement [18].

All analyses were performed with the statistical programming language R (R Core Team 2019) (R version 4.2.2 (09 March 2024) [19].

The study was conducted in accordance with the Declaration of Helsinki and approved by the Ethics Committee for ethical approval (EK 313/13-University Hospital Bonn).

## 3. Results

### 3.1. Patient and Disease Characteristics

The study included 92 PwMS, of which 64 (69.6%) were female and 28 (30.4%) male. Of these, 9 PwMS (9.8%) showed the primary progressive, 69 PwMS (75.0%) showed the relapsing–remitting, and 14 PwMS (15.2%) showed the secondary progressive form of MS. Bladder emptying was spontaneous in 89 cases (96.7%); in 1 case (1.1%), it required triggering; and it was conducted in 2 cases (2.2%) by residual urine catheterisation after spontaneous micturition. The descriptive analysis of the demographic variables is shown in Table 1.

Of the 92 PwMS, 20 (21.7%) had no urological symptoms at the time of the analysis, 3 (3.3%) had symptoms lasting 1–6 months, 1 (1.1%) for 6–12 months, 15 (16.3%) for 1 to 2 years, 15 (16.3%) for 3 to 5 years, 12 (13%) for 6–10 years, and 5 (5.4%) over 10 years. Overall, 21 PwMS (22.8%) did not specify the duration of urological symptoms. An overview of the distribution and type of urological symptoms is shown in Table 2.

### 3.2. Interrater Reliability of the Suspected Diagnoses

#### 3.2.1. Analysis A

**Figure 2:**
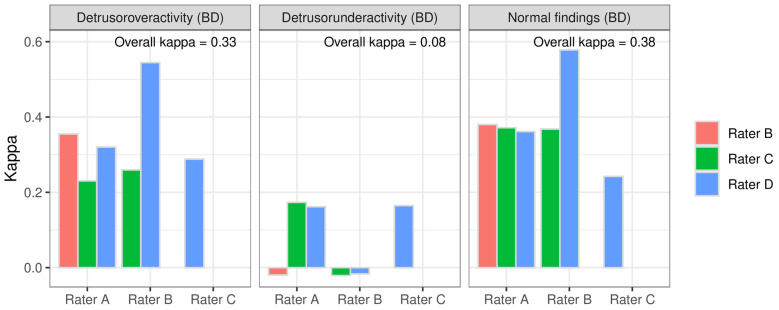
Interrater reliability of single suspected diagnoses from anamnesis and bladder diary between the raters paired and overall.

**Figure 3:**
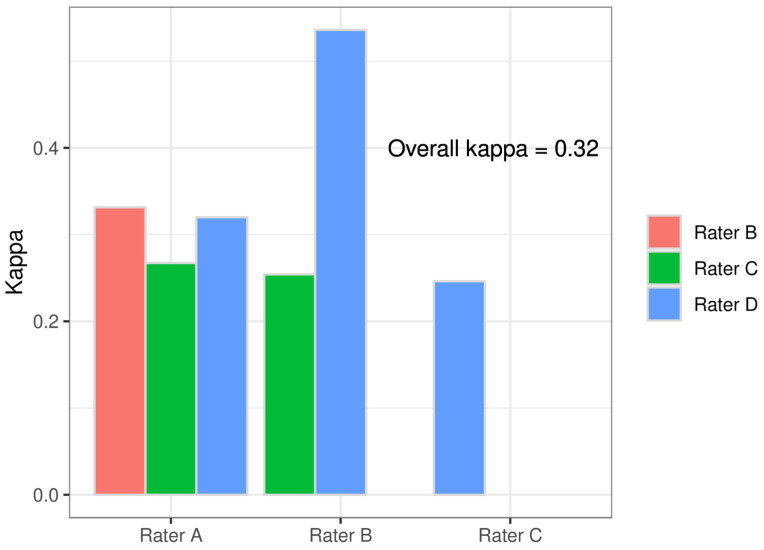
Interrater reliability of all suspected diagnoses from anamnesis and bladder diary between the raters paired and overall.

For the suspected diagnosis from medical history and BDs, the raters had 3 options: normal findings (NF), detrusor overactivity (DO) and detrusor underactivity (DU) for single selection. The paired comparisons for each singular suspected diagnosis were as follows: for NFs, κ_C_ = 0.24 to κ_C_ = 0.58; for DO, κ_C_ = 0.23 to κ_C_ = 0.54; and for DU, κ_C_ = −0.02 to κ_C_ = 0.17 (Figure 2). The paired comparison between the raters for all suspected diagnoses showed Cohens’ kappa values of 0.25 to 0.54 and thus fair-to-moderate agreement [18] (Figure 3). The results of the comparison of all raters (Fleiss’ kappa) are shown in Figure 2 and Figure 3. DU achieved the lowest IRR.

#### 3.2.2. Analysis B

**Figure 4:**
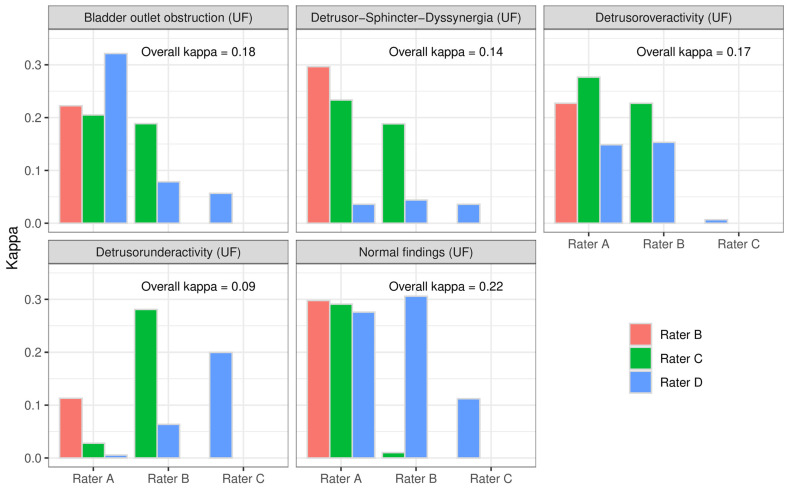
Interrater reliability of the single suspected diagnoses between the paired raters and overall (analysis B-1).

The raters had 5 options for the suspected diagnosis from medical history, BDs, and UF. In addition to NFs, DO, DU, bladder outlet obstruction (BOO), and detrusor–sphincter–dyssynergia (DSD) could be selected. This resulted in multiple-choice answers and thus 14 combinations for suspected diagnoses. The IRR was notably lower for combinations compared to the individual diagnoses with a maximum κ_C_ = 0.19. Figure 4 shows the paired interrater reliability of the single suspected diagnoses (analysis B-1). As in analysis A, the diagnosis DU achieved the lowest IRR. Table 3 shows the paired interrater reliability of the combinations for suspected diagnoses (analysis B-2).

A uniform consensus with 100% agreement between all raters was found for the diagnoses NO, DU, BOO, and DSD in one data set each and for DO in 8 data sets.

### 3.3. Interrater Reliability of the Therapy Suggestions (Analysis C)

**Figure 5:**
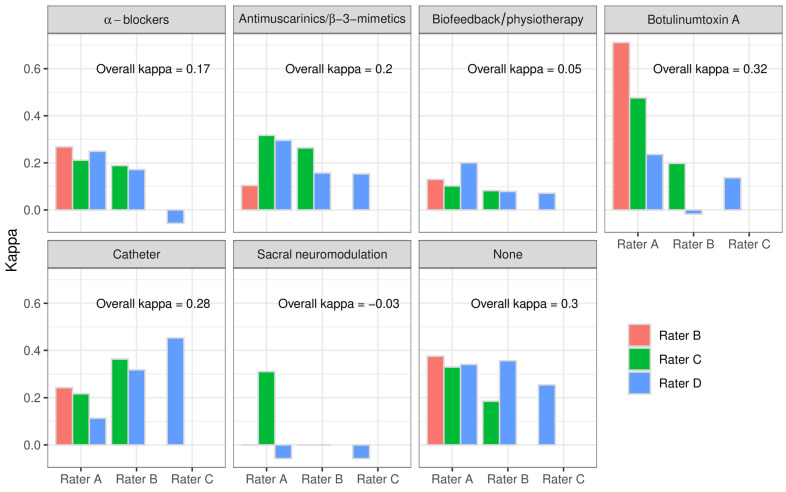
Interrater reliability of the single therapy suggestions between the raters paired and overall.

The IRR of the therapy suggestions from UF was determined to be analogous to the suspected diagnoses for each of the seven predefined therapy suggestions and for the therapy combinations that occurred. For the several treatment suggestions considered, NO κ_C_ = 0.38, KAT κ_C_ = 0.45, and BTX κ_C_ = 0.71 showed the highest IRR between the rater pairs. We revealed the lowest IRR for NM κ_C_ = −0.06 and P/BF κ_C_ = 0.07 (Figure 5).

The possibility of therapy combinations resulted in a variety of selected combinations. The combined treatment suggestions showed a remarkably low agreement with a Fleiss’ kappa of 0.08. The best agreement in this subanalysis was achieved by the therapy suggestion “no therapy” with a κ_F_ = 0.31. Table 4 provides an overview of the IRR between the rater pairs for combinations of treatment suggestions.

## 4. Discussion

In everyday urological practice, it is often impossible to examine PwMS regarding NLUTD using video urodynamics. Although the examination represents the gold standard in the diagnostics of NLUTD [7], it is invasive, associated with side effects [20], costly, and limited in availability [21]. Compared to persons with traumatic or congenital damage of the spinal cord, NLUTD in PwMS results in less damage to the upper urinary tract [22] and not every PwMS is initially affected by NLUTD [23]. Therefore, the rationalised work-up of the NLUTD in PwMS is continually the subject of various expert panels and guideline recommendations [23]. However, a standardised recommendation and predictor for the presence of NLUTD—reviewed in prospective longitudinal studies—have not yet been clearly identified. At the same time, multiple sclerosis results in a high financial and personnel burden on the healthcare system [24] and leads to a severe loss in the quality of life of those affected [23]. To address all those needs, it is essential to develop a sufficient and less costly screening/preselection procedure [9]. In light of these facts, there is a growing interest in UF as a non-invasive, straightforward urodynamic procedure [1,6]. UF is widely available in urological practices and together with medical history and BDs it is often the basis of treatment decisions in daily practice. Therefore, the aim of our work was to analyse the IRR of UF not only regarding the suspected diagnosis, but also the derived clinical consequences. To this end, we had 4 neuro-urologically experienced raters assess 92 data sets based on medical history, BDs, and UF from PwMS regarding predefined suspected diagnoses and therapy suggestions.

For the diagnoses from medical history, BDs, and UF, we obtained a fair agreement with kappa values of 0.32 (κ_C_) and 0.2 (κ_F_), respectively, for the singular consideration of the diagnoses, both between the rater pairs and in the overall rater comparison. However, many kappa values were lower, especially when combinations of diagnoses were considered. For the singular analyses of the treatment suggestions, the kappa values tended to be slightly higher. Particularly in the treatment suggestions “catheter”, “botulinum toxin” and “none”, the kappa between the rater pairs was up to 0.71 and was 0.32 in the overall comparison of all raters. When looking at treatment combinations, the IRR fell significantly to values below 0.2 for slight agreement. The fewer options were available, the higher was the raters’ agreement.

This is a methodological problem: the more choices available to raters, the lower is the probability of deciding for the same result. This is particularly the case when the decisions are not dichotomous, e.g., pathological or non-pathological, but are based on an individual interpretation by the rater. Gacci et al. [16], involving over 100 urologists specialised in functional urology, were able to show that there is a high IRR when curves and numerical uroflowmetry results are categorised as normal or abnormal. When a diagnosis was included, the IRR in the same group of investigators fell to a κ value of up to <0.1, except for the diagnosis “no abnormalities”, where substantial agreement (0.7) was still achieved. This could be because the decision in favour of or against a therapy is still based on a dichotomous decision-making process. The study by van de Beek et al. [25] showed analogous results as early as 1997, whereby the diagnosis “normal findings” also showed the best IRR.

In paediatric urological studies, the IRR of UF also showed similar results. Chang and Yang, 2008, showed a regularly substantial κ (0.68–0.81) in the differentiation of normal vs. pathological findings; in the assessment of specific, pathological waveforms, the IRR was κ = 0.07 for the same investigators [26]. As soon as an interpretative aspect was included in the diagnosis, the IRR dropped considerably.

The study by Faasse et al. [15] also focussed on the assessment of the UF curve shape in children. The IRR of uroflow EMG was analysed and a specific selection of diagnoses was specified in the study design. Working according to fixed diagnostic criteria, this was a single selection. An agreement of κ = 0.33 up to a maximum of 0.74 was demonstrated between the rater pairs. The slightly lower kappa values observed in our work in comparison to those reported in the studies mentioned before can be attributed to the fact that, in addition to the querying specific curve shapes, several diagnostic suggestions may also be applicable. In our study, the storage function of the bladder could be assessed using normal findings, detrusor overactivity, and detrusor underactivity, as well as the micturition phase with DSD or BOO. This resulted in many combinations, despite the predefined answers in the form. However, these answers reflect the complexity of the diagnostic and decision-making process that results from UF in daily practice: a clear idea of the underlying diagnosis is essential to determining treatment recommendations. A simple yes/no decision as to whether a pathological condition exists is not sufficient.

The low IRR for DU was noticeable in all our results. On the one hand, we believe that this is due to the poor differentiability between DU and BOO in UF for both genders [27,28,29,30], as the shape of the curve and the measured uroflow parameters can be identical for both diagnoses. On the other hand, the clinical definition and diagnosis of DU and detrusor hypocontractility [30,31,32,33,34] are still not finally clarified. Additional indices and parameters are constantly being tested [28,29,31] in order to reliably differentiate the diagnosis of DU with non-invasive measures. These aspects have a negative influence on the consistency of the raters, which influences κ_C_ [14]. Another aspect of information that has an impact on κ_C_ is the prevalence of a trait. The less frequently a characteristic is actually present or the less frequently it is estimated to be present, the lower κ_C_ is [14]. We are unable to assess the influence of the prevalence of the diagnoses in our current study at this time, as we have not correlated the diagnoses with the urodynamic results. This will be the subject of a further study in which the IRR of UD and UF will be compared.

In summary, various authors consider the diagnostic value of UF to be insufficient [13] or as limited [15,16,17] due to the IRR achieved. Some authors consider UF to be a good screening tool between normal and abnormal micturition [26]. However, all authors see the need for standardisation in the reporting of the curves [15,16,17,26].

In our opinion, in its current form UF can be an adjunct to the management of NLUTD in PwMS, e.g., act as a follow-up during therapy compared to a pre-treatment assessment. However, the design of all previous studies on IRR does not allow the conclusion that having a limited IRR is also detrimental to the affected patient. The reasons for decisions are not included and could provide reasonable explanations for different decisions. Each rater has an individual horizon of experience and follow-up strategies. The IRR would need to be combined with an outcome measure of treatment success to be able to make statements about the question of unfavourable treatment success with a low IRR.

In the future, artificial intelligence-supported, learning algorithms based on UD studies could be used to refine the diagnostic value of UF for PwMS. These would need to be designed to allow dichotomous decisions to be made as a small detail of the whole, leading to a more complex diagnostic or therapeutic decision. Parameters with a high degree of selectivity and impact on the therapy decision must be identified for this purpose. The first artificial intelligence-assisted approaches to the diagnosis of detrusor underactivity in men are already available [32]. Choo et al. have developed algorithms for the reporting of uroflowmetry in men and women using artificial intelligence [35]. However, the curve so far can only be assessed as normal or pathological, and the results are compared with the judgements of urological specialists and not with underlying urodynamics. Incorporating uroflowmetry with other clinical predictors, working according to the methodology demonstrated by Ito et al. [36], could offer another comprehensive approach to estimating the necessity for additional diagnostics for NLUTD in PwMS. Therefore, further investigations are needed to correlate the results of UD with non-invasive diagnostics and clinical treatment outcomes in PwMS.

## 5. Conclusions

The suspected suggested diagnosis and treatment suggestions from UF are subject to a high level of individual influence by the raters. If there are several differential diagnoses and therapies to choose from, as it occurs in the daily use of UF, interrater reliability decreases considerably. This results in a variety of possible treatment decisions. To evaluate the possible long-term relevance of UF in addition to UD, the current gold standard in diagnostics of NLUTD in PwMS, UD should be subjected to the same analysis. The results of this analysis then should be compared with those of the UF. According to our study, the standardisation of UF findings based on a dichotomous decision-making algorithm could help to improve the consistency of the assessment.

## 6. Limitations

Our study had some limitations. The main limitation was that the findings were based on medical records. The raters did not have the real patients in front of them. The influence of the patient’s constitution, such as physical or mental limitations, and the effect on the decision of the rater was therefore completely absent. Furthermore, the data originate from a neurological inpatient rehabilitation clinic, which has an influence on the type of PwMS participating, as more severe or advanced cases requiring rehabilitation are usually treated in these institutions (selection bias). In summary, all raters had the same conditions, and the focus of this study was on interrater reliability and not on the most correct result for the patient.

## Figures and Tables

**Table 1:** Descriptive analysis of the demographic variables.

	Mean (SD)	Median (IQR)	Min; Max	Missing N (%)
Age of patients in years	47.5 (9.4)	48 (40.8; 55.2)	25; 72	0 (0)
Duration of MS in months	118.7 (96.7)	102.5 (35.8; 168.2)	1; 445	0 (0)
Expanded Disability Status Scale	3.9 (1.4)	4 (2.9; 4.5)	1,5; 8	12 (13.04)

**Table 2:** Overview of the urinary symptoms of the included PwMS (number of PwMS (N) = 92, no missings).

Neurogenic Lower Urinary Tract Symptoms	Yes N (%)	No N (%)
Staged micturition/interrupted urine flow	18 (19.6)	74 (80.4)
Initiation delay	11 (12)	81 (88)
Weak urine stream	11 (12)	81 (88)
Residual urine sensation	18 (19.6)	74 (80.4)
Desire to press	15 (16.3)	77 (83.7)
Drizzle	5 (5.4)	87 (94.6)
Urinary retention	1 (1.1)	91 (98.9)
Nocturia	37 (40.2)	55 (59.8)
Pollakiuria	45 (48.9)	47 (51.1)
Urgency	52 (56.5)	40 (43.5)
Incontinence	50 (54.3)	42 (45.7)

**Table 3:** Overview of interrater reliability of combined suspected diagnoses between the raters paired and overall (analysis B-2).

Paired Interrater Reliability of the Combined Suspected Diagnoses			
	Match rate in % Cohen’s kappa 95% CI		
Rater	B	C	D
A	30.4% 0.19 0.09; 0.28	22.8% 0.13 0.04; 0.22	23.9% 0.14 0.06; 0.23
B		22.8% 0.12 0.03; 0.21	21.7% 0.1 0.01; 0.19
C			25% 0.09 0; 0.18
Fleiss’ kappa 0.12			

**Table 4:** Overview of the interrater reliability between the rater pairs for combinations of treatment suggestions (analysis C-2).

Paired Interrater Reliability of the Combined Therapy Suggestions			
	Match rate in % Cohen’s kappa 95% CI		
Rater	B	C	D
A	15.2% 0.09 0.02; 0.16	17.4% 0.1 0.03; 0.16	19.6% 0.1 0.02; 0.18
B		17.4% 0.09 0.02; 0.17	10.9% 0.05 0.0; 0.11
C			17.4% 0.1 0.03; 0.17
Fleiss’ kappa 0.08			

## Data Availability

The data presented in this study are available on request from the corresponding author. The data are not publicly available due to privacy.
